# Macaque Cardiac Physiology Is Sensitive to the Valence of Passively Viewed Sensory Stimuli

**DOI:** 10.1371/journal.pone.0071170

**Published:** 2013-08-05

**Authors:** Eliza Bliss-Moreau, Christopher J. Machado, David G. Amaral

**Affiliations:** 1 Department of Psychiatry and Behavioral Sciences, University of California Davis, Sacramento, California, United States of America; 2 California National Primate Research Center, Davis, California, United States of America; 3 The M.I.N.D Institute, University of California Davis, Sacramento, California, United States of America; 4 Center for Neuroscience, University of California Davis, Davis, California, United States of America; Centre national de la recherche scientifique, France

## Abstract

Autonomic nervous system activity is an important component of affective experience. We demonstrate in the rhesus monkey that both the sympathetic and parasympathetic branches of the autonomic nervous system respond differentially to the affective valence of passively viewed video stimuli. We recorded cardiac impedance and an electrocardiogram while adult macaques watched a series of 300 30-second videos that varied in their affective content. We found that sympathetic activity (as measured by cardiac pre-ejection period) increased and parasympathetic activity (as measured by respiratory sinus arrhythmia) decreased as video content changes from positive to negative. These findings parallel the relationship between autonomic nervous system responsivity and valence of stimuli in humans. Given the relationship between human cardiac physiology and affective processing, these findings suggest that macaque cardiac physiology may be an index of affect in nonverbal animals.

## Introduction

Variation in autonomic nervous system activity is observed during a wide variety of psychological processes ranging from cognitive control to social processing to emotion (e.g., [1]–[5]). Popularized by the works of James [6] and Cannon [7], the autonomic nervous system (ANS) has played a central role in theories of emotion for more than a century. While specific autonomic nervous system markers of discrete emotions such as fear and happiness have not been identified, patterns of physiological responding do appear to be related to the positive or negative valence of experience [8]. In humans, there is a substantial history of monitoring affective responding during passive viewing tasks in which people look at still photos or videos that vary in their positive and negative content (e.g., [5], [8]–[11], for a review, [12]). Often, measures of ANS activity are used to index induced affective states in such passive viewing tasks (e.g., [5], [13]; for reviews see [14], [15]). The goal of the present study was to investigate whether rhesus macaques (*Macaca mulatta*) show psychophysiological responses that mirror those observed in humans as they passively watch dynamic affective visual stimuli. Rhesus macaques are a particularly good model species for human biological and psychosocial processing because their physiology, neurobiology, and complex social structure are similar to that of humans (for reviews [16], [17]).

Cardiac function is modulated by the sympathetic and the parasympathetic branches of the ANS [18]–[20]. Global measures of ANS activity, such as heart rate, are regulated by the sympathetic and parasympathetic nervous systems together [21] and inconsistently track psychological states. Heart rate, for example, can be elevated either during states of anger or happiness, and either elevated or depressed during fear (see [14] for a review). Without ample contextual information and other behavioral or self-report measures, global measures of ANS activity are only useful for determining that the state of the organism has changed, not for determining precisely *how* it has changed. In contrast, selective measures of autonomic nervous system activity - those that selectively index sympathetic or parasympathetic activity - provide more fine-grained information about what aspects of the ANS are shifting and are arguably more closely related to discrete psychological states. Pre-ejection period (PEP) and respiratory sinus arrhythmia (RSA) provide selective measures of sympathetic and parasympathetic activity, respectively [21].

Pre-ejection period, a measure of ventricular contractility, is the length of time between the heart being signaled to beat and the opening of the aortic valve. As sympathetic activity increases, ventricular contractility also increases leading to a shorter PEP. In humans, PEP is consistently shorter during negative, as compared to positive, emotional experiences. In other words, sympathetic activation is greater during negative affective states than positive affective states [8]. To our knowledge, there are no published reports on pre-ejection period as an index of psychological processing in nonhuman primates.

RSA is a measure of the variation in time between heart-beats during inhalation and exhalation that is mediated by vagal (parasympathetic) efferent pathways [22]. RSA is, therefore, respiration-related heart rate variability. Low RSA has been associated with anxiety [23], [24], depression [25], and worrying [26]. Momentary task related decreases in RSA have been documented during tasks that are stressful or have high attentional demands (e.g., [27], [28]; [20], [29] for reviews). Conversely, RSA is higher during positive experiences. One study [30], found that resting RSA is highest for people who report the highest levels of positive affect and rewarding social interactions.

Few studies have measured RSA in nonhuman primates, but those that have find a similar pattern of responding in macaques as in humans. RSA in long-tailed macaques (*Macaca fascicularis*) was significantly lower during the sounding of a startling whistle [31], the presentation of a capture glove [31], or during introduction to a novel environment [32] than when no negative stimulus was present. While these findings suggest that macaque RSA may be lower during negative experiences, RSA in response to positive affective content has not yet been explored.

It is worth noting that other studies in macaques have measured physiological channels associated with affective processing. There are two limitations to the extant literature. First, many of the physiological measures used reflect a blend of sympathetic and parasympathetic nervous system activity. For example, heart rate (e.g., [33]–[37]) and nasal temperature (e.g., [38], [39]) have been measured to index affective processing in macaques. Second, even in studies that do use specific measures of parasympathetic [31], [32] or sympathetic [38], [40] responsivity, the number and type of stimuli to which animals are exposed is very limited (e.g., a single capture glove [31] or a single 10-second video clip of a novel monkey threatening the camera; [38]). As a result, it is difficult to draw conclusions about how the autonomic nervous system, generally, and the parasympathetic and sympathetic branches, specifically, contribute to a broad range of affective states.

Given their psychosocial and anatomical similarities, we hypothesized that similar patterns of sympathetic and parasympathetic responding to stimuli with varying affective properties would be observed in rhesus macaques, as in humans. We hypothesized that sympathetic nervous system activity would increase and parasympathetic nervous system activity would decrease across a set of stimuli that ranged from positive to negative. To test this hypothesis, we showed adult male rhesus macaques a set of 300 30-second videos showing unfamiliar conspecifics engaging in behaviors that varied in terms of their affective valence (ranging from very negative to very positive). Videos of conspecifics are particularly potent stimuli insofar as they capture macaque attention (e.g. [41]) and can hold it for long durations of time (e.g., [42]). Further, macaques generate species typical behaviors in the presence of videos suggesting that they find their content realistic (e.g., [43]–[46]). We also evaluated a number of additional indices of psychological and social processes (e.g., social status information depicted in the video clip) that are potentially related to ANS activity. Our goal was to control for the possible psychological processes that might influence autonomic responsivity in order to specifically examine the impact of valence on sympathetic and parasympathetic responsivity.

## Materials and Methods

This study was carried out in strict accordance with the recommendations in the Guide for the Care and Use of Laboratory Animals of the National Institutes of Health. Experimental procedures were approved by the University of California, Davis Institutional Animal Care and Use Committee (Protocol Number 13483). All testing occurred at the California National Primate Research Center (*CNPRC*). Every possible effort was undertaken to minimize animals' stress and promote their wellbeing.

### Subjects and Living Conditions

Subjects for this study were four adult male rhesus macaques (age range: 5.8 to 8.7; weight range 10 to 14 kg) that were part of a larger study on visual attention [41]. Subjects were born and raised, until at least two years old, in large outdoor field cages at the CNPRC, measuring 0.2 ha and housing ∼60–100 animals each. Upon relocation indoors, subjects were housed in standard (according to NIH guidelines for caging sized based on weight) adult macaque laboratory cages (66 cm wide×61 cm long×81 cm high) and paired with a compatible animal. Pairing occurred either for a minimum of 6 hours per day, 5-days a week, or 24 hours per day, either via full access (allowing both animals access to both cages during the pairing time) to through a metal grate (allowing tactile access). At the time of this study, all subjects had a male pair-mate and also had visual and auditory access to other animals in the housing room.

The housing room was maintained on a 12-hour light and dark cycle, with lights turning on at 6 am and turning off at 6 pm. Animals were fed monkey chow (Lab Diet #5047, PMI Nutrition International INC, Brentwood, MO) twice daily, provided with fresh fruit and vegetables twice per week, and had access to water *ad libitum*. In addition, subjects received the standard enrichments given to all macaques at the CNPRC— a rice/oat/pea mixture on their forage boards once per day, a rubber Kong toy or large Nylabone in their cages at all times, fresh coconuts once per month, and periodic delivery of fruit and vegetables in puzzle balls or puzzle tubes. Animals were not sacrificed at the end of the experiment.

### Experimental Protocol

Information regarding animal training, experimental stimuli, equipment, and the eye-tracking study are detailed elsewhere [41], [47]. Briefly, all animals were trained to sit in a modified primate chair (Crist Instrument Co., Inc., Damascus, MD), to have their head restrained using thermoplastic helmets, and their arms and feet gently restrained using leather straps (1.3 cm×3 mm×1 m). Testing occurred in a sound attenuated chamber (Acoustic Systems, Austin, TX; 2.1 m×2.4 m×1.1 m). The test chamber included: a video eye-tracker (Applied Science Laboratories, Bedford, MA; model R-HS-S6; 53.34 cm from the animals' eyes); a wide-screen, color monitor (60.96 cm diagonal; Gateway Inc., Irvine, CA; model LP2424) positioned at the animals' eye level (127 cm from the animals' eyes); physiological recording equipment (MindWare Technologies, Ghanna, OH); and an automatic juice dispenser (Crist Instrument Co., Inc.; model # 5-RLD-E3) with curved mouthpiece (Crist Instrument Co., Inc.; model # 5-RLD-00A) that attached to the top-left of the chair. A white noise generator (60 dB) masked auditory distractions.

**Figure 1:**
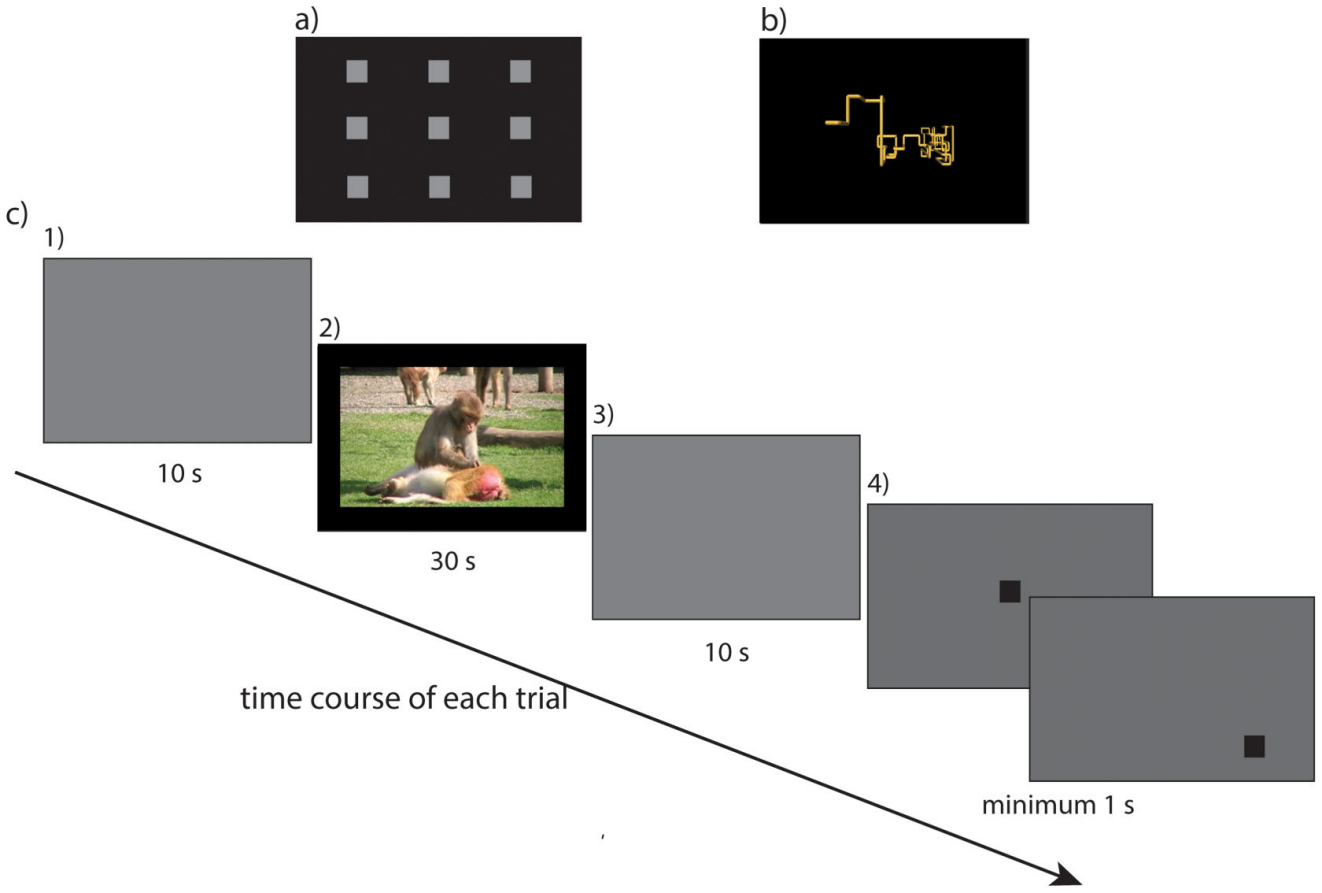
Experimental Procedure. a) Eye-tracker calibration. b) Test chamber acclimation phase. c) Experimental phase. d) Schematic of electrode placement on monkeys. e) Examples of the ECG signal overlaid onto a cardiac impedance signal for one ensemble 30-second movie.

Testing occurred over 12 test days during which animals saw 300 social videos (content listed in Table 1). Each video was viewed only once by each monkey. Each daily test session consisted of three phases (depicted in Figure 1, panels a through c). First, the eye tracker was calibrated for each animal by having him fixate on small video stimuli presented in nine different positions on the screen (Figure 1a). Animals next completed a test-chamber acclimation phase during which they watched 10, 30-second videos of PC screensavers in order to allow them to acclimate to being in the test chamber and ensure that the eye tacking calibration was accurate (Figure 1b).

**Table 1:** Specific social behaviors depicted in the 30-second videos.

Social Video Content	Examples of stimuli	Filming location (if applicable)
Aggression	Group aggression on one monkeyOne-on-one fightingLengthy threats exchanged between monkeys	Field enclosures
Aggressive facial displays and body postures	ThreatsTooth-grindingCage shaking	Plexiglass test cage
Submissive/affiliative facial displays and body postures	LipsmackingPresenting rumpCowering	Plexiglass test cage
Neutral facial displays and body postures	Monkeys with neutral faces and bodies	Plexiglass test cage
Nonspecific social content	Monkeys walking around cagesMonkeys sleeping together	Field enclosures
Foraging	Monkeys foraging in gravelMonkeys eating chow	Field enclosures
Grooming	One-on-one groomingGroup grooming	Field enclosures
Mounting	Animals mounting each other, includes males mounting females, males mounting males, and females mounting females	Field enclosures
Play	Rough and tumble play, typically between young animals	Field enclosures

Note: Specific examples of content are indicated in the second column. Filming location: The content for social videos was filmed by CJM and laboratory staff at the CNPRC either at the large field cages or in a laboratory test cage equipped with a clear Plexiglass front.

During the *experimental* phase, each trial included the same sequence of stimuli: 1) a grey screen with luminance equal to the average luminance of the trial's video (10 s); 2) a video (30 s; 720×480 resolution; visual angle: 23.2^0^×12.6^0^; 3) a grey screen with luminance equal to the average luminance of the trial's video (10 s); 4) a 50% grey screen with a black square (3.4^0^ visual angle) at its center; 5) a 50% grey screen with a black square (3.4^0^ visual angle) positioned at one of eight points around the periphery of the screen. Monkeys were required to fixate on the black squares (for a minimum of 500 ms) in order to advance to the next trial, thus ensuring their maintained attention and the calibration of the eye-tracker. Successful fixation resulted in juice reward (180 ms juice delivery for center target; 360 ms peripheral target). Each test day included 25 videos with social content, intermixed with 25 videos with content from nature documentaries [41] not relevant to the present report. Social videos were filmed at the California National Primate Research Center. Each movie included content that, to the greatest extent possible, reflected only one type of social behavior. None of the videos included footage of humans, non-macaque nonhuman primates, snakes, or predators of macaques. Video presentation order was pseudorandomized and fixed across test days. The baseline and experimental phases were presented via E-Prime 2.0 (Psychology Software Tools, Pittsburgh, PA).

### Video Ratings

Each video was rated in terms of six psychological variables to characterize its psychological properties. Specifically, the content of each video was rated for its valence, arousal, dominance, submission, proximity of animals interacting, and the number of unique social interactions. See Table 2 for a description of each index and its rating scale. Raters were six members from our laboratory who were familiar with macaque behavior and had between one and ten years of experience working with macaques. Raters were explicitly instructed that the videos varied a good deal in terms of their content and that their task was to provide “summary” judgments of each full 30-second clip. Ratings were then averaged across raters to create a composite index score for each video; Cronbach's alphas were computed for each index (all were above .9). Ranges and variances are presented in Table 2.

**Table 2:** Psychological content variables, definitions, and characteristic ranges with variances.

Variable	Definition	Scale	Composite Score Range (Variance)
*Affective Properties*			
Valence	How negative or positive is the depicted content?	(−3) Extremely negative(−2) Moderately negative(−1) Mildly negative(0) Neutral(1) Mildly positive(2) Moderately positive(3) Extremely positive	−2.80 to 2.40 (1.96)
Arousal	How activating (“ramped-up”) or deactivating (relaxed/calming) is the video content?	(−3) Extremely deactivated(−2) Moderately deactivated(−1) Mildly deactivated(0) Neutral(1) Mildly activated(2) Moderately activated(3) Extremely activated	−2.60 to 2.80 (2.20)
*Characteristics of Social Interactions*			
Dominance	Did animals exhibit behaviors related to dominance?	(0) No dominance(1) Mild dominance(2) Moderate dominance(3) Extreme dominance	0 to 3.00 (0.52)
Submission	Did animals exhibit behaviors related to submission?	(0) No submission(1) Mild submission(2) Moderate submission(3) Extreme submission	0 to 2.4 (0.44)
Proximity of Animals	Are animals in proximity or far away from each other?	(−3) Extremely far(−2) Moderately far(−1) Slightly far(0) One animal only(1) Slightly close(2) Moderately close(3) Extremely close	−.80 to 3.00 (1.39)
Number of Animal Interactions	How many unique interactions occur between animals?	(0) No interactions(1) Few interactions(2) Moderate number of interactions(3) Many interactions	0 to 3.00 (1.18)

Note: Distribution of videos by valence is as follows: −2.8 through −2.0 (N = 36); −1.8 through −1.0 (N = 10); −.80 through −.2 (N = 20); 0 (N = 31); 0.2 through 0.8 (N = 84); 1.0 through 1.8 (N = 59); 2.0 through 2.4 (N = 60).

### Psychophysiological Data Collection and Processing

Cardiac physiological data were collected noninvasively using standard silver/silver chloride, pre-gelled snap surface electrodes (Bio-Detek Incorperated, Pawtucket, RI) and MindWare Technology hardware and software (MindWare Technologies, Gahanna, OH). Electrode sites on the animals were shaved with a battery-operated clipper, cleaned with gauze soaked in 70% ethanol, and allowed to air dry. Cardiac impedance electrodes were attached in a configuration identical to that used in humans [48] (placement on the notch of the clavicle and xyphoid process on the animal's front side, and ∼3 cm above and below the front electrodes on the back surface). ECG electrodes were attached in a modified lead II configuration. Once electrodes were affixed to the animals' skin, the animals' torsos were wrapped with an elastic disposable bandaging material (4 inch Coban Self-Adherent Wrap, 3M Health Care, St. Paul, MN) in order to keep electrodes in place during the testing session.

Physiological signals were recorded at 1000 Hz. Cardiac impedance was measured by passing a small current (0.4 mA) between the outer electrodes and measuring, via the inner electrodes, basal impedance resulting from changes in blood volume and distribution (Zo) and its first derivative (dZ/dt). Physiology data were subjected to standardized scoring procedures using commercially available software (MindWare HRV3.0.1 and IMP3.0.6, MindWare Technologies, Gahanna, OH). Cardiac impedance cycles were ensembled over each 30-second epoch. B and X points were adjusted manually. PEP was the time between the ECG's Q-point (initiation of left ventricle contraction) and the dZ/dt's B point (opening of the aortic valve). RSA was computed from the ECG with the Zo as the respiration signal using HRV 3.0.1 software according to accepted scoring parameters [49]. The ECG signal for each 30 s epoch was visually inspected to ensure proper placement of the R-points and artifacts were removed or corrected. The data were detrended, tapered, and fast Fourier transformed. RSA was computed as the natural log integral of the high frequency power (0.24 to 1.04 Hz). The high frequency band was set to 0.24 to 1.04 based on previous literature in small children with similar heart and respiration rates [50]. This band encompassed the respiratory frequency of all animals.

### Eye-Tracking Data Collection and Processing

Eye-tracking data were previously collected for the purposes of a study of visual attention that has already been reported [41]. These data were used in the present study, however, to control for individual differences in attention devoted to each of the 300 videos of interest. For nonverbal subjects such as rhesus monkeys, the only way to objectively infer visual attention is through foveal gaze location and duration [51]. We therefore chose to use the total number of fixations and the total duration of fixations directed at the videos as an index of visual attention. To collect these measures, we used a video-eye tracker from Applied Sciences Laboratories (ASL) and processed the data with the associated ASL software using the default settings. Eye tracking indices were computed for each video for a single area of interest that covered the entire video frame. The onset of a fixation was recorded when gaze coordinates remained within a 1°×1° visual angle for 100 milliseconds and terminated when the coordinates left that space for more than 360 milliseconds.

### Data Analysis Strategy

Data were subjected to Hierarchical Linear Modeling using HLM 6.08 [52]. Experimental videos (level-2) were nested within monkey (level-1). The level-2 intercept term was allowed to vary (i.e., it included an error term) to account for individual differences in cardiac responding between animals. This allowed us to use raw data, rather than requiring us to normalize the psychophysiological data for each animal based on its responsivity during the baseline phase. Properties of the experimental videos detailed in Table 2 (e.g., average valence) were predictors of either RSA or PEP. The effects of the independent variables were fixed in level-2. Psychological content variables were added into the model using raw scores (see Table 2) because a “0” score was *a priori* defined as either the mid-point of the scale or the low extreme of the scale. In addition to testing the main effects of the psychological content variables, we also probed all possible two-way interactions between the psychological content variables. Visual inspection of the distribution of dominance information over the valence continuum indicated that videos that scored extremely high in dominance were exclusively negative. We therefore selected a final model that included the valence X dominance interaction term to assess the impact of dominance information on the psychological variables. Models were estimated using restricted maximum likelihood.

To account for the possibility that varied attention to the stimuli, rather than valence per se, may be driving the variation in physiological responding, we also completed an additional *post hoc* set of analyses in which we used previously reported visual attention metrics computed from eye-tracking data [41] as additional predictors of PEP and RSA. To test this possibility, we computed two new models each for PEP and RSA. In the first, total fixation frequency was added to the existing model for PEP and the existing model for RSA (i.e., that included the valence X dominance interaction term). In the second model, total fixation duration was added to the existing model for PEP and the existing model for RSA.

## Results

### The impact of valence and other psychological content on physiological responding

**Figure 2:**
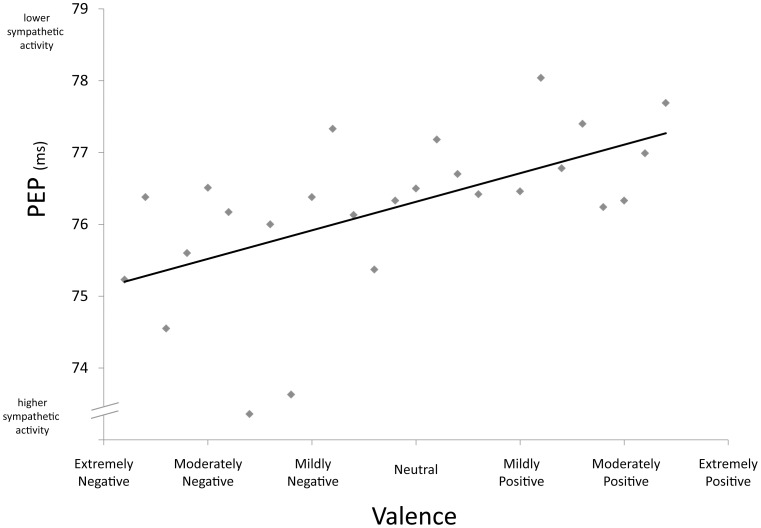
PEP by valence. Each data point represents the mean PEP (in milliseconds) for videos of a given average valence score across the four subjects. The regression line is based on the coefficients in Table 3. It depicts the influence of valence on PEP controlling for all other psychological variables. The Y-axis has been repositioned for ease of interpretation; the intercept occurs when valence is neutral (scored value of 0).

**Figure 3:**
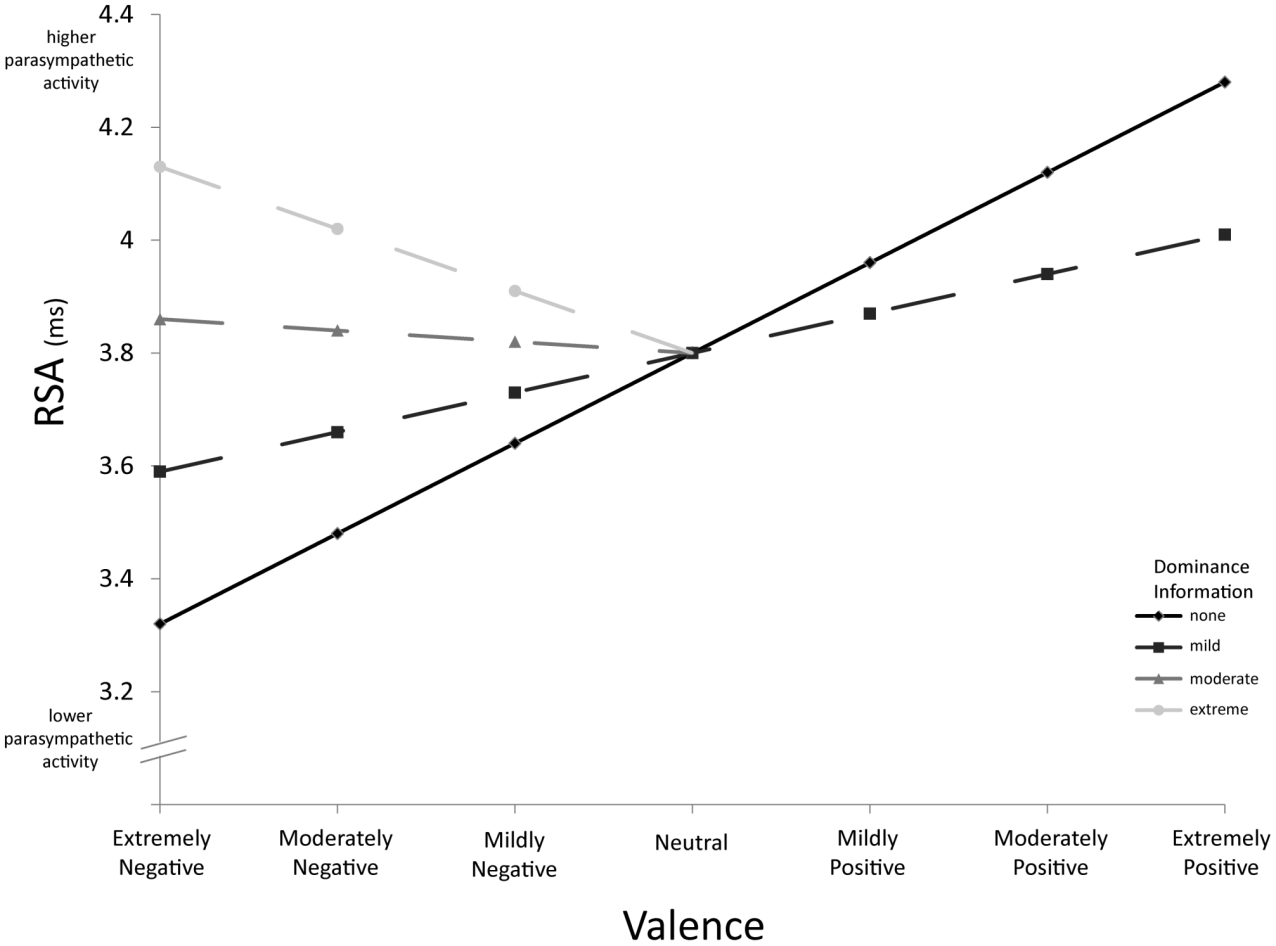
Predicted values of RSA by valence and dominance. The solid line depicts the effect of valence on RSA controlling for all other variables. The dotted lines depict the effect of valence on RSA at different levels of dominance information. Note that moderate and extreme levels of dominance information were not present in videos rated to be positive. The Y-axis has been repositioned for ease of interpretation; the intercept occurs when valence is neutral (scored value of 0).

As hypothesized, the valence of the videos predicted both PEP and RSA. As video content changed from negative to positive, sympathetic activity decreased (see Figure 2; Table 3) and parasympathetic activity increased (Figure 3, see Table 4). These findings map precisely onto the patterns of sympathetic and parasympathetic reactivity reported in humans.

**Table 3:** Sympathetic Activity (PEP) as Predicted by Psychological Content.

Predictor	Coefficient	SE	*t*	*df*	*p*
Intercept, γ_00_	76.40	6.90	11.07	3	<.0005
**Valence, γ_10_**	**0.44**	**0.20**	**2.25**	**1168**	**0.024**
Arousal, γ_20_	0.21	0.14	1.51	1168	0.132
Dominance, γ_30_	0.19	0.40	0.47	1168	0.640
Submission, γ_40_	−0.10	0.25	−0.39	1168	0.696
Proximity, γ_50_	0.22	0.19	1.12	1168	0.264
**Interactions, γ_60_**	**−0.46**	**0.23**	**−2.03**	**1168**	**0.043**

Note: Dependent variable is PEP. Bolded font indicates significant predictor variables.

**Table 4:** Parasympathetic Activity (RSA) as Predicted by Psychological Content.

Predictor	Coefficient	SE	*t*	*df*	*p*
Intercept, γ_00_	4.31	0.34	12.00	3	<.0005
**Valence, γ_10_**	**0.13**	**0.05**	**2.90**	**1167**	**0.004**
Arousal, γ_20_	0.01	0.03	0.18	1167	0.858
Dominance, γ_30_	0.01	0.09	0.07	1167	0.948
Submission, γ_40_	0.02	0.04	0.42	1167	0.677
Proximity, γ_50_	0.03	0.03	0.89	1167	0.375
**Interactions, γ_60_**	**−0.09**	**0.04**	**−2.06**	**1167**	**0.040**
**Valence X Dominance, γ_70_**	**−0.07**	**0.03**	**−2.18**	**1167**	**0.029**

Note: Dependent variable is RSA. Bolded font indicates significant predictor variables.

Beyond valence, sympathetic and parasympathetic responses were also sensitive to psychological variables such as the number of animal interactions depicted in the video clip. Controlling for other psychological variables (i.e., those listed in Table 2; e.g., valence, dominance, etc.), sympathetic activity increased and parasympathetic activity decreased as the number of animal interactions depicted in the video increased (Tables 3 and 4). Parasympathetic, but not sympathetic, activity was also influenced by dominance information (Figure 3). When social interactions in the videos were very negative, those that did not include clear information about the dominance relationships between animals generated lower parasympathetic responses than those that did include clear dominance information. For example, an aggressive interaction in which two animals were fighting and displaying similar aggressive behaviors generated a lower parasympathetic response than an aggressive interaction in which there was a clear aggressor.

### The impact of visual attention on physiological responding

To examine the possibility that differential attention (as indexed by fixation frequencies and duration) to positive and negative video stimuli might be driving the impact of valence on sympathetic and, or, parasympathetic responding, we added both total fixation frequency per video and total fixation duration to the HLM models predicting PEP and RSA. Neither total fixation frequency nor total fixation duration were significant predictors of PEP (total fixation frequency: *coefficient* = 0.0089, *SE* = 0.012, *t*(1167) = 0.72, *p* = .0472; total fixation duration: *coefficient* = −0.026, *SE* = .0043, *t*(1167) = −0.61, *p* = .0541). Most importantly, controlling for the visual attention parameters did not alter the relationship between valence and PEP (total fixation frequency: *coefficient* = 0.48, *SE* = 0.20, *t*(1167) = 2.36, *p* = 0.019; total fixation duration: *coefficient* = 0.45, *SE* = 0.20, *t*(1167) = 2.26, *p* = 0.024) or the relationship between the number of animal interactions and PEP (total fixation frequency: *coefficient* = −0.52, *SE* = 0.24, *t*(1167) = −2.15, *p* = 0.032; total fixation duration: *coefficient* = −0.46, *SE* = 0.23, *t*(1167) = −2.03, *p* = 0.042). Interestingly, none of the other psychological variables significantly predicted sympathetic responsivity after controlling for visual attention.

As in the analyses for sympathetic responsivity, controlling for the impact of visual attention on parasympathetic responding did not dramatically alter the impact of the significant psychological predictors. Valence remained a significant predictor when controlling for both fixation frequency and fixation duration (total fixation frequency: *coefficient* = 0.10, *SE* = 0.05, *t*(1166) = 2.07, *p* = 0.038; total fixation duration: *coefficient* = 0.13, *SE* = 0.04, *t*(1167) = 2.95, *p* = 0.004). The valence X dominance interaction's impact on RSA dropped to a trend level effect when controlling for total fixation frequency (*coefficient* = −0.06, *SE* = 0.03, *t*(1166) = −1.73, *p* = 0.084), but remained significant when controlling for total fixation duration (*coefficient* = −.07, *SE* = 0.03, *t*(1166) = −2.3, *p* = 0.026). Finally, the impact of the number of animal interactions on RSA was no longer significant when controlling for total fixation frequency (*coefficient* = −0.05, *SE* = 0.04, *t*(1166) = −1.04, *p* = 0.301), but remained significant when controlling for total fixation duration (*coefficient* = −0.09, *SE* = 0.04, *t*(1166) = −2.09, *p* = 0.036). Interestingly, however, there was a significant effect of visual attention on RSA indicating that as visual attention increased, parasympathetic activity decreased. As both fixation frequency and fixation duration increased RSA decreased (total fixation frequency: *coefficient* = −0.006, *SE* = .0002, *t*(1166) = −2.64 *p* = 0.009; total fixation duration: *coefficient* = −0.02, *SE* = 0.008, *t*(1167) = −2.35, *p* = 0.019).

## Discussion

The findings from this experiment demonstrate that passively viewed dynamic visual stimuli evoke predictable changes in rhesus monkey autonomic activity. Both sympathetic and parasympathetic activity varied in monkeys as they observed video stimuli that ranged from negative to positive. The pattern of effects in the experimental monkeys was similar to that which has previously been observed in humans ([8], [20], [29] for reviews). This suggests that cardiac autonomic responsivity may play a similar role in affective responding in humans and nonhuman primates. Aspects of the social context depicted in the video stimuli accounted for variance in both sympathetic and parasympathetic responding above and beyond the variance explained by valence alone, suggesting that autonomic responsivity encodes more than the hedonic properties of stimuli. The extent to which these effects hold during actual social interactions (e.g., being aggressed upon or being aggressive, interacting with a dominant animal) is an avenue for future research.

When many animals were present in a video, and if there was little or no information about the positive or negative nature of their interactions or the dominance relationship between individuals, sympathetic activity increased and parasympathetic activity decreased in the observer monkey - possibly as a precursor to potential fight or flight. These findings are consistent with the perspective that increases in parasympathetic activity support pro-social affiliative behaviors while decreases in parasympathetic activity are associated with experience or perception of aversive, dangerous or stressful social situations (cf. [22]). An alternative, complementary, explanation is that a situation in which there is the possibility of many social interactions but the specific nature of those interactions is unknown requires greater cognitive control (or mental effort) on the part of the observer to fully process or interpret. This perspective is supported by findings indicating that high cognitive control is associated with relatively increased sympathetic and decreased parasympathetic activity (e.g., [2]).

The idea that ambiguous social situations require increased cognitive control resulting in an altered course of physiological responding may also explain the impact of dominance information and valence together on parasympathetic activity. We found that when stimuli were negative and dominance information was unclear, parasympathetic responding decreased as compared to when stimuli were negative and dominance information was very clear. Quick perception and accurate evaluation of dominance-related behaviors is critical to survival for macaques whose social groups are characterized by a dominance hierarchy that is maintained largely through aggression. Dominance perception may be particularly important to male macaques, such as our subjects, that migrate from their natal groups and therefore do not inherit their dominance rank and must establish it on their own [53], [54]. It is reasonable to expect that monkeys invest significant cognitive control into assessing the dominance status of conspecifics and the dominance/submission relationships in groups of animals. In this view, greater cognitive control is required when dominance information is unclear, driving down parasympathetic responding. In contrast, when dominance information is very clear in the context of negative interactions, parasympathetic activity can increase because thus monkeys know which other animals are “in charge” (and thus, who to avoid in the current occasion and potentially who to befriend on future occasions). Critically, regardless of how animal number or dominance information effect ANS responsivity, the main effect of valence on ANS responsivity held when controlling for the number of animals present in the video and the dominance information that they communicated. Future studies of the impact of dominance information in visual stimuli and dominance status of the subject animals will help us to understand the dynamic relationship between dominance and cardiac responsivity, and in particular, allow us to investigate why dominance information was related to parasympathetic but not sympathetic processing in this experiment.

By evaluating the effect of visual attention on sympathetic and parasympathetic responding we were able to rule out the possibility that variation in objective measures of attention alone were driving the effects of valence on psychophysiology. Whether using fixation frequency or duration as our indicator of visual attention, the impact of valence on both sympathetic and parasympathetic responding held. Further, visual attention was not a significant predictor of sympathetic activity, nor did controlling for it alter the impact of any of the other psychological variables on sympathetic activity. In contrast, visual attention did significantly predict parasympathetic activity as might be expected based on the human literature. As visual attention increased, parasympathetic activity decreased. This finding is similar to those demonstrating that tasks that require greater cognitive resources depress parasympathetic activity more than tasks that require fewer resources (e.g., [1]–[3]). This finding further supports the translational value of cardiac psychophysiological indices. Investigating why visual attention is related to parasympathetic but not sympathetic activity is a potentially fruitful avenue of future research.

Future research should also address the limitations of the present study (e.g., the subjective ratings of the videos and the small sample size). For example, while the present sample size is typical for nonhuman primate studies, it would be important to test additional animals in the future that have different temperamental characteristics (e.g., low versus high anxiety). It would also be fruitful to test a wider variety of subjects—female animals, animals that vary in age, animals that vary in socialization history, etc.—to evaluate whether the patterns of psychophysiological responding depicted here are generalizable to a more diverse sample. Similarly, the findings that dominance information alters the parasympathetic response to social stimuli points to an interesting, testable hypothesis about the impact of an animal's own dominance ranking on his or her physiological responsivity to dominance information. This hypothesis could be tested by evaluating physiological responding from a larger group of animals that vary in their dominance ranking. The noninvasive nature of our methods make each of these potential studies eminently feasible with the nonhuman primate.

Translational neuropsychological and behavioral neuroscience methods that attempt to map macaque behaviors onto human psychological states have largely relied upon behavioral observations that require human observers to catalog behaviors and then imbue those behaviors with meaning. These approaches assume that the same affective state underlies behaviors that are physically homologous, or occur in similar contexts, across species. While behaviors such as a “grimace” are typically interpreted as reflecting negative experience or submission [55] and often discussed as relating to human “fear” [56], grimaces occur in a wide variety of contexts including those which are likely positive or appetitive (e.g., during orgasm, [57]) and when animals signal that they understand social relationships in the absence of any other affective change [58]. Clearly, objective measures of nonhuman primate affect would go some way towards establishing a firm basis for comparison of human and animal affect and the mechanisms that underlie it. In this paper, we have demonstrated that cardiac psychophysiological measures provide a noninvasive, objective measure of macaque affective processing, in real-time with high temporal resolution. The present work represents a step forward in understanding and monitoring the affective processing of nonhuman primates within a conceptual and methodological framework that can be directly replicated in humans. As such, this work allows for the advancement of translational methods investigating basic psychological processes and disease models.
